# Unveiling genome sequence of *Bacillus safensis* strain WOB7 KX774196, a linamarin-utilizing bacterium (LUB) isolated from cassava wastewater (CWW) in Lagos State, Nigeria

**DOI:** 10.1128/mra.00247-24

**Published:** 2024-06-25

**Authors:** Adewale K. Ogunyemi, Olanike M. Buraimoh, Wadzani P. Dauda, Olufunmilayo O. Akapo, Bukola C. Ogunyemi, Titilola A. Samuel, Matthew O. Ilori, Olukayode O. Amund

**Affiliations:** 1 Department of Microbiology, University of Lagos, Akoka, Lagos State, Nigeria; 2 Department of Biological Sciences (Microbiology Unit), Lagos State University of Science and Technology, Ikorodu, Lagos State, Nigeria; 3 TETFund Centre of Excellence on Biodiversity Conservation and Ecosystem Management (TCEBCEM), University of Lagos, Akoka, Lagos State, Nigeria; 4 Department of Agronomy (Crop Science Unit), Federal University, Gasuha, Yobe State, Nigeria; 5 Department of Biochemistry and Microbiology, University of Zululand, KwaDlangezwa Main Campus, KwaDlangezwa, 3886, South Africa; 6 Department of Biochemistry, University of Lagos, Idi-Araba, Lagos State, Nigeria; Portland State University, Portland, Oregon, USA

**Keywords:** *Bacillus safensis*, linamarin-utilizing bacterium, whole-genome sequence, cassava wastewater, cyanogen, detoxify

## Abstract

*Bacillus safensis* strain WOB7 is a linamarin-utilizing bacterium (LUB) that was isolated from cassava wastewater obtained from a processing factory. We present here the draft genome sequence of the strain (WOB7). These data provide valuable information on the prospects of the linamarase and other genes of importance associated with cyanogen detoxification.

## ANNOUNCEMENT

**Fig 1:**
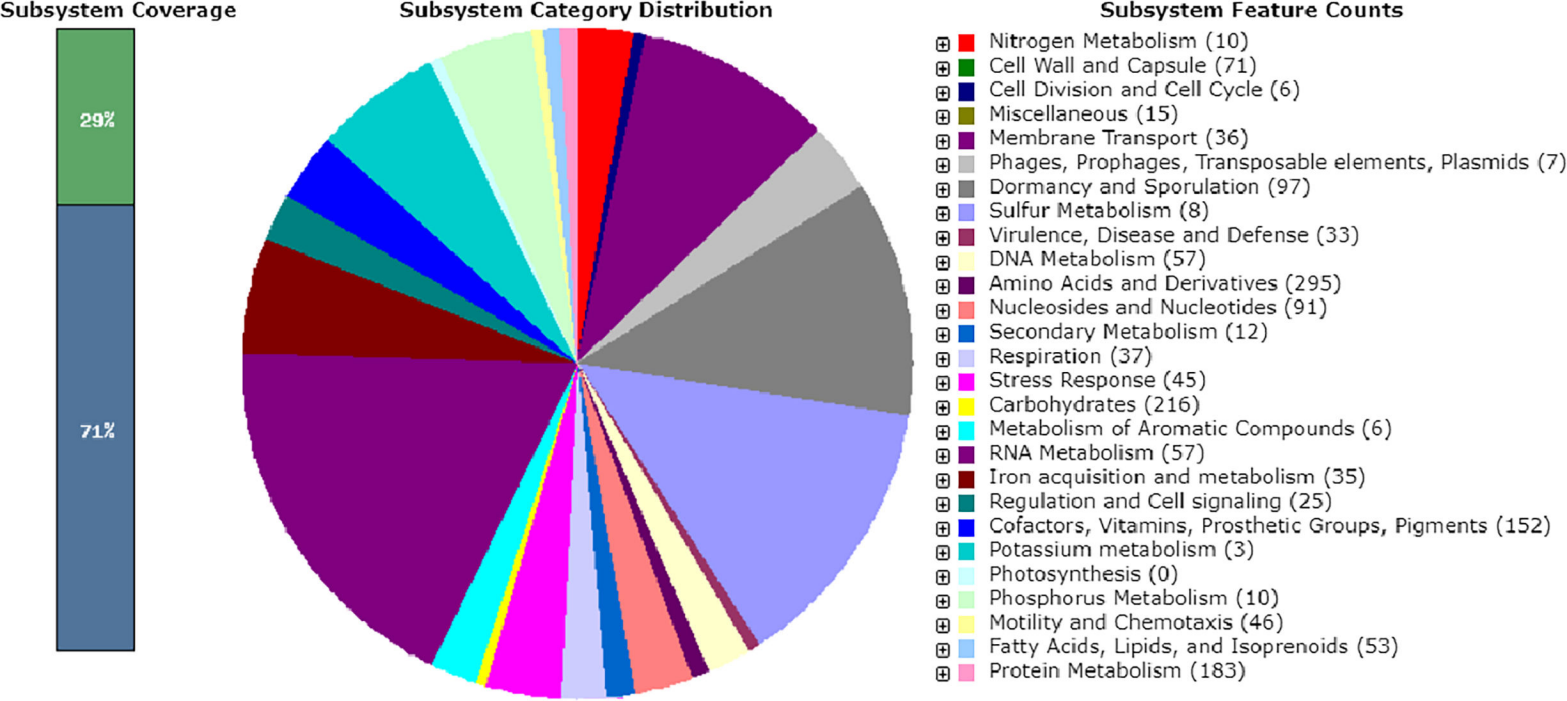
The RAST analysis-based subsystem distribution of whole-genome sequence of *Bacillus safensis* strain WOB7. Each colour in the pi graph represents a particular group of genes mentioned on the right site of the graph.

Cassava production has globally increased by 240 million metric tons. By 2025, 62% of global cassava production is predicted to come from Sub-Saharan Africa (1). Nigeria, as the world’s largest producer, generates a significant amount of cassava waste (1). Cassava mill wastewater is the liquid waste generated during cassava processing. It contains high organic content, linamarin (a cyanogenic glucoside that produces hydrocyanic acid (HCN)), minerals (2 3), and has an acidic pH (4). Therefore, it poses a significant threat to the environment and the health of communities living near cassava mills (5). Here, we report the draft genome of strain WOB7, a type strain of *Bacillus* s*afensis*, a member of the Bacillaceae isolated from cassava wastewater obtained from a cassava processing factory in Odogunyan, Ikorodu, Lagos State, Nigeria (6°39′39.40236″E3°30′33.19883″). The CWW sample was mixed, serially diluted with sterile water, and then cultured on nutrient agar (NA) plate. After aerobic incubation at 37°C for 24 h, small and white colonies were picked out and then restreaked on the NA plate until pure cultures were obtained. Strain WOB7 was identified as *B. safensis* KX774196, using the universal primers 27F and 1492R by 16S rRNA gene sequencing (6). The strain was grown on nutrient broth medium for 24 h at 37 °C for DNA extraction. A single colony was inoculated in 5 mL of nutrient broth medium and incubated at the same condition (24 h at 37°C) to obtain cell biomass. Afterward, 2 mL of the culture was centrifuged (16,000 × *g*, 4 min), and the pellet was extracted for genomic DNA with the Zymo Research Fungal/Bacterial DNA MiniPrep Kit (catalog no. D4300; Zymo Research Corp., Irvine, CA, USA), according to the manufacturer’s instructions. Then xGen DNA library preparation kit was used for 2 × 150 bp paired-end sequencing with the Illumina HiSequencing platform (HiSeq4000X). The obtained raw reads from each run were quality-checked using FastQC (v.1.0.0, BaseSpace Illumina) and subsequently trimmed using FastXToolkit (v.2.2.5, BaseSpace Illumina) (7). Genome *de novo* assembly and assembly quality were performed using SPAdes (v.3.9.0, BaseSpace Illumina) (8, 9). The draft genome sequences were annotated using the NCBI Prokaryotic Genome Annotation Pipeline (v.6.6), with default parameters (10). Also, the genome annotation and metabolic pathway identification were carried out by RAST (v.2.0) server (11). Default parameters were used except where otherwise noted. Table 1 shows the genomic features of *Bacillus safensis* WOB7. The genome of strain WOB7 consists of 15,185,963 reads and 75 contigs, with GC content of 41.5%. A total of 3.7 × 10^6^ bp (3.7 Mb) genomic size was generated, corresponding to 569.5X genome coverage. Figure 1 shows the RAST analysis-based subsystem distribution of the whole-genome sequence of strain WOB7. These genomic data add information to clarify the *B. safensis* metabolic strategies to detoxify cassava wastewater.

**TABLE 1:** Genome features of *Bacillus safensis* strain WOB7^*a*^

Attribute	Value
Total reads	15,185,963
Genome coverage (X)	569.5
Genome size (Mb)	3.7
CDSs (total)	3,744
CDSs (with protein)	3,711
CDSs (without protein)	33
Genes (total)	3,836
Number of contigs	75
Contig N50 (kb)	149.5
Contig L50	8
GC content (%)	41.5
tRNA	71
rRNA	4,10, 2
Genes (RNA)	92
ncRNA	5
Accession number	JAYSGV010000000

^*a*^ CDS, coding sequence; GC, guanine-cytosine content; ncRNA, non-coding RNA; rRNA, ribosomal RNA; tRNA, transfer RNA.

## Data Availability

A whole-genome sequence of *Bacillus safensis* strain WOB7 has been deposited in GenBank under accession number JAYSGV010000000, BioProject number PRJNA1057587, BioSample number SAMN38321375, and Sequence Read Archive accession number SRX24013597.
