# Erratum for Ogunyemi et al., “Unveiling genome sequence of *Bacillus safensis* strain WOB7 KX774196, a linamarin-utilizing bacterium (LUB) isolated from cassava wastewater (CWW) in Lagos State, Nigeria”

**DOI:** 10.1128/mra.00806-24

**Published:** 2024-08-30

**Authors:** Adewale K. Ogunyemi, Olanike M. Buraimoh, Wadzani P. Dauda, Olufunmilayo O. Akapo, Bukola C. Ogunyemi, Titilola A. Samuel, Matthew O. Ilori, Olukayode O. Amund

## ERRATUM

Volume 13, no. 7, e00247-24, 2024, https://doi.org/10.1128/mra.00247-24. Page 3, Author Affiliations: Wadzani P. Dauda’s affiliation should read “Department of Agronomy (Crop Science Unit), Federal University Gashua, Gashua, Yobe State, Nigeria.”

